# Addressing Learning Needs on the Use of Metagenomics in Antimicrobial Resistance Surveillance

**DOI:** 10.3389/fpubh.2020.00038

**Published:** 2020-02-25

**Authors:** Ana Sofia Ribeiro Duarte, Katharina D. C. Stärk, Patrick Munk, Pimlapas Leekitcharoenphon, Alex Bossers, Roosmarijn Luiken, Steven Sarrazin, Oksana Lukjancenko, Sünje Johanna Pamp, Valeria Bortolaia, Jakob Nybo Nissen, Philipp Kirstahler, Liese Van Gompel, Casper Sahl Poulsen, Rolf Sommer Kaas, Maria Hellmér, Rasmus Borup Hansen, Violeta Munoz Gomez, Tine Hald

**Affiliations:** ^1^Research Group for Genomic Epidemiology, National Food Institute, Technical University of Denmark, Lyngby, Denmark; ^2^SAFOSO AG, Bern-Lieberfeld, Switzerland; ^3^Department of Infection Biology, Wageningen Bioveterinary Research, Lelystad, Netherlands; ^4^Faculty of Veterinary Medicine, Institute for Risk Assessment Sciences, Utrecht University, Utrecht, Netherlands; ^5^Veterinary Epidemiology Unit, Faculty of Veterinary Medicine, Ghent University, Merelbeke, Belgium; ^6^Department of Health Technology, Technical University of Denmark, Lyngby, Denmark; ^7^Research Group for Microbiology and Hygiene, National Food Institute, Technical University of Denmark, Lyngby, Denmark; ^8^Intomics A/S, Lyngby, Denmark

**Keywords:** surveilance, metagenomics, MOOC, antimicrobial resistance, one health

## Abstract

One Health surveillance of antimicrobial resistance (AMR) depends on a harmonized method for detection of AMR. Metagenomics-based surveillance offers the possibility to compare resistomes within and between different target populations. Its potential to be embedded into policy in the future calls for a timely and integrated knowledge dissemination strategy. We developed a blended training (e-learning and a workshop) on the use of metagenomics in surveillance of pathogens and AMR. The objectives were to highlight the potential of metagenomics in the context of integrated surveillance, to demonstrate its applicability through hands-on training and to raise awareness to bias factors^1^. The target participants included staff of competent authorities responsible for AMR monitoring and academic staff. The training was organized in modules covering the workflow, requirements, benefits and challenges of surveillance by metagenomics. The training had 41 participants. The face-to-face workshop was essential to understand the expectations of the participants about the transition to metagenomics-based surveillance. After revision of the e-learning, we released it as a Massive Open Online Course (MOOC), now available at https://www.coursera.org/learn/metagenomics. This course has run in more than 20 sessions, with more than 3,000 learners enrolled, from more than 120 countries. Blended learning and MOOCs are useful tools to deliver knowledge globally and across disciplines. The released MOOC can be a reference knowledge source for international players in the application of metagenomics in surveillance.

## Introduction

The dissemination of knowledge on antimicrobial resistance (AMR) is, like AMR itself, a global, transversal challenge, and needs to be tackled in collaboration between the public health, veterinary and food systems, i.e., in a One Health or integrated approach. A One Health AMR surveillance is challenged by the need to coordinate between surveillance programmes, distinct for each sector. It is therefore important to develop harmonized methods for detection of AMR determinants across sectors (1). In Europe, several initiatives are contributing to the development of integrated AMR surveillance, including the European Epidemiologic Network (Epi-NET),^2^ the European Union Joint Programming Initiative on Antimicrobial Resistance (JPIAMR)^3^, the Joint Interagency Antimicrobial Consumption and Resistance Analysis (JIACRA)^4^ and the EU One Health Action Plan against AMR^5^.

The development of integrated surveillance depends on the definition of AMR itself and the choice of a quantitative measure that can be used for comparisons within and between different target populations. AMR can be defined based on established phenotypic thresholds (i.e., interpretation of minimum inhibitory concentration (MIC) or inhibition zone according to specific guidelines [e.g., CLSI and EUCAST]) and based on gene-centric definitions (2). Traditional AMR surveillance relies on the monitoring of phenotypic AMR in indicator organisms (e.g., *Escherichia coli*) and selected pathogens (e.g., serotypes of *Salmonella enterica* subsp. *enterica*), while in metagenomic studies the definition of AMR is gene-centric.

Recent studies have shown that gene-centric AMR monitoring using whole genome sequencing (WGS) of isolates can be highly concordant with observed phenotypic resistance (3–6), although at different levels of accuracy between antibiotic classes. Gene-centric approaches allow to differentiate whether AMR is due to the presence of acquired resistance genes or due to mutations in chromosomal genes, and to identify genes embedded into mobile genetic elements, which are transferable among bacteria.

Although such findings encourage the implementation of WGS in AMR monitoring (7), WGS remains a culture-based method, which challenges the production of real-time actionable information.

Shotgun metagenomics is the culture-independent, untargeted sequencing of all DNA present in a sample, and it therefore offers the possibility to investigate taxonomic composition (including viable and non-viable, culturable, and non-culturable organisms), to predict microbial functions (including AMR) and to recover whole genome sequences (8) (which may reveal yet undiscovered reservoirs of ARGs). A gene-centric, culture-independent method, such as metagenomics allows monitoring AMR with a common measure across surveillance programs, which is independent of the choice of sector-specific indicator- and pathogenic- organisms. Indicator organisms, such as *E. coli*, have often been selected due to convenience and scalability, and not necessarily for being the most appropriate organism to monitor overall AMR trends in a microbial community. Furthermore, it is possible with metagenomics to investigate interactions between species in a microbial community (9) which may determine the occurrence of resistant organisms. Finally, it also has the potential to overcome infrastructure limitations hampering reliable AMR surveillance in low- and middle-income countries, since it requires less tightly controlled environmental conditions and less diversified laboratory supplies compared to traditional microbiology methods (10). Finally, metagenomics surveillance yields data in a standardized format that can be stored and shared electronically with overall modest investments.

There are however shortcomings and biasfactors that need to be taken into account when applying metagenomics (11). The results may be biased due to sampling (including the sample matrix) (9, 12), and the community composition can be affected by sample handling (12, 13). Furthermore, DNA extraction (12, 14, 15), sequencing library preparation (16), the sequencing technology (17, 18), the bioinformatics approach (19), the reference databases used (2), and downstream statistical analyses (20) may also bias results. Finally, there are concerns related to the low sensitivity of metagenomics, which probably constitutes the main obstacle to its application in AMR surveillance. There is an obvious need for benchmarking studies targeting different steps of the process and it is essential to be aware of the importance of method validation and protocol harmonization.

AMR surveillance is a complex topic under rapid scientific development, and the potential to embed new methods into policy in the future calls for an appropriate knowledge dissemination strategy. Open online education (e-learning) is an effective, flexible, and cost-efficient way to disseminate knowledge to a large and diverse range of target learners, at a global level. The delivery of online courses has been greatly facilitated by web-based platforms that host massive open online courses (MOOCs), generally offered free of charge (21). Blended learning, i.e., a mix of training delivery formats, allows for the combination of traditional conceptual lectures delivered through e-learning with face-to-face sessions of hands-on work with tutor support^1^. This facilitates learning in topics where practical data analysis and data interpretation are relevant, and additionally facilitates discussions and networking between course participants.

There are several internationally available MOOCs covering the topics of antimicrobial resistance (21),^6^^,^^7^^,^^8^^,^^9^^,^ genomics^10^^,^^11^^,^^12^^,^^13^^,^^14^ or One Health^15^. However, to the best of our knowledge, there are no current initiatives to provide information and training on the use of metagenomics in the context of AMR surveillance, particularly in a transdisciplinary way (i.e., covering topics from sampling strategy to data analysis).

The goal of the European project Ecology from Farm to Fork Of Microbial drug Resistance and Transmission (EFFORT) is to provide scientific evidence on the epidemiology and consequences of AMR in the food chain, while implementing metagenomics^16^ (22, 23). Within the scope of EFFORT, we developed a blended training programme on the use of metagenomics in surveillance of pathogens and AMR to (1) Highlight the potential of metagenomics in a global, integrated surveillance context, (2) Demonstrate its applicability by providing hands-on training on a surveillance case-study, and (3) Raise awareness for the factors that may bias metagenomics results^1^. The training consisted of an e-learning component delivered 1 month ahead of a one-and-a-half-day hands-on workshop. After the workshop, we re-evaluated and revised the e-learning, before its stand-alone launch as a MOOC^1^.

## Pedagogical Framework

The blended training programme consisted of an e-learning component and a one-time face-to-face workshop. The resources used for development of lectures and practical exercises included peer-reviewed scientific publications and the instructors' own expertise. The instructors' background included a variety of disciplines, such as bioinformatics, microbiology, epidemiology, and veterinary medicine^1^. The target group of learners included staff of competent authorities responsible for AMR monitoring (i.e., veterinary services, food safety authorities and reference laboratories), as well as academic staff^1^.

The development of the training was led by the Research Group for Genomic Epidemiology at the National Food Institute, Technical University of Denmark (DTU FOOD), which is the EU reference laboratory for antimicrobial resistance (EURL-AR) and comprises multidisciplinary expertise relevant to metagenomics-based AMR surveillance. The objective was to cover the different stages of the workflow in metagenomics-based surveillance, providing the learners with a practical overview of how to conduct each step^1^. Individual lectures from all instructors were subject to peer-review, to avoid overlaps and ensure message consistency.

## Programme Development And Delivery

### Pedagogical Format

#### E-Learning

The online course was originally organized in “four modules intended to be delivered over 4 weeks, with a separate graded assessment after each module. The modules were: (1) *Introduction*, (2) *From sampling to sequencing*, (3) *From reads to results*, and (4) *Potential of metagenomics for surveillance*. On average, the expected learning time per week was 2 h” minimum ^1^.

The course was implemented and delivered in the platform Coursera^17^, which gathers e-learning courses from the world's top universities and education providers ^1^. Before its delivery to the workshop participants, it was offered to a private group of volunteers, in order to gather feedback. The e-learning was released 1 month before the workshop. The e-learning component was subsequently revised and adapted to a MOOC, with the title “Metagenomics applied to surveillance of pathogens and antimicrobial resistance,” and it is freely available at https://www.coursera.org/learn/metagenomics. On Coursera, public courses run in 4-weeks sessions, and learners in the same session work through the course together. Sessions start automatically on a regular schedule, and enrolment for each session opens and closes automatically^1^.

Table 1 summarizes the course structure and content, as it is presently available online. E-learning elements include video lectures, in-video quizzes, complementary reading, case-study reports and module assessment quizzes. “Lectures are delivered in English, with English subtitles, and pdfs from every lecture are available from the course page. In most videos, non-graded quizzes are included to ensure the engagement of the learners in the lecture and consolidate the learning of key concepts. Reading elements are provided as a complement to most lectures to reinforce the knowledge transmitted, and eventually provide additional information on the topic. Also, a glossary of the terms used during the course is provided in the first module”^1^.

**Table 1:** MOOC structure and content and corresponding learners' feedback (accessed 31/01/2020).

**Module**	**Elements**	**Topic**	**Lecture**	**Likes**	**Dislikes**
	1 lecture 2 readings		Welcome lecture	97	2
From sampling to sequencing	9 lectures 9 readings	Introduction to metagenomics and antimicrobial resistance	Introduction to Metagenomics	72	
			Considerations and controls for metagenomic/microbiome projects	52	
			Introduction to antimicrobial resistance	49	
		Sampling and sample handling	Sampling for surveillance	38	
			Sampling at farms and slaughterhouses	30	2
			Sample storage	19	1
		DNA and RNA extraction methods	Isolation of DNA from complex samples	27	
			Sample processing for viral metagenomics	11	1
		Sequencing	Notes on library preparation	11	
			Sequencing platforms	29	
Module 1 assessment	2 quizzes 2 readings			59	3
From reads to results	6 lectures 5 readings	Bioinformatics concepts and tools for metagenomics analysis	General intro to bioinformatics analysis of metagenomics data	24	3
			Overview of available metagenomics analysis tools	23	5
			MG mapper	35	1
			ResFinder database	20	
			Demo of metagenomic classification using KRAKEN	12	1
			Real example of metagenomic analysis–lessons learned	13	1
Module 2 assessment	1 quiz 6 readings			25	1
Interpretation of results and potential of metagenomics for surveillance	5 lectures 6 readings	Interpretation of results and application of metagenomics in surveillance	Virtual machine setup	5	
			Analysis and visualization of read count data	12	
			Metagenomic assembly and binning–reconstructing genomes from reads	23	1
			Application of metagenomics in surveillance–methods	20	
			Application of metagenomics in surveillance–opportunities and challenges	15	
Module 3 assessment	1 quiz 3 readings			13	1
Final assessment	5 quizzes 7 readings			23	8
	1 lecture		Farewell lecture	9	

*Likes/dislikes for each topic include lecture videos and corresponding reading(s), or all elements of a module assessment*.

The course assessment is divided in three module-specific graded multiple option quizzes and a final quiz. Each module quiz includes “questions to assess the theoretical knowledge obtained in the corresponding module, and questions based on a surveillance case-study, transversal to the overall course”^1^. The case-study material includes an outline of the exercise step at each module, and module-specific reports for interpretative analysis. “Quiz questions are presented in a multiple-choice format, some with a single correct answer, and others with multiple correct options. In order to complete a module successfully, the learners are required to answer 80% of the quiz correctly”^1^.

The final assessment quiz includes questions which require hands-on work by the learners, similarly to what was required to the workshop participants. This is expected to improve the active learning potential of the MOOC. Tutorials for the different steps of the final quiz (*virtual machine setup, introduction, sampling, quality control, bioinformatics analysis of metagenomics results* and *analysis of metagenomics results in a surveillance context*) are provided as additional course elements.

#### Workshop

Part of the workshop program was based on a recapitulation of the e-learning and the remaining consisted on new content, particularly hands-on training, with exercise sessions following a case-study. Workshop lectures were complemented with discussion sessions, which were distributed throughout the programme in order to foster the exchange of impressions among participants. Two quizzes, at the beginning and at the end of the workshop, were used in order to collect the background information of the participants, their feedback on the training and their opinion on the use of metagenomics for AMR surveillance. A report on the blended training is available at the EFFORT website^1^.

The participants worked in groups during the exercises. “A virtual machine (including user guide) was built for the purpose of the workshop to make use of specific software” ^1^, including FastQC (24) for quality control, MGmapper (25) for read classification and R (26) for read count analysis and epidemiological analysis. The participants were also introduced to and had the opportunity to apply Linux command-line. They were provided with fictional metagenomics and epidemiological data of a hypothetical case-study in order to perform the analyses. Teaching materials are publicly available at Metagenomics Training Report^1^.

### Learning Environment

The e-learning was first delivered in a pilot session to a group of 14 volunteers from the EFFORT consortium to gather feedback before launching. After launching, it was delivered to a group of 155 registered learners, including all workshop participants^1^.

“A total number of 41 participants and 7 speakers from 14 countries attended the workshop”^1^. Most participants had a research and microbiology background, and were employed at University (52%) or at a Government research institute (32%). Competent authorities (5%) and the Industry (5%) were also represented among participants. The two top reasons for registering on the workshop were “a general interest in the topic” and “a continuing education for the current job.” These were followed by “informing current research” and “continuing education for a future job.”

By January 2020, 52.0% of the MOOC enrolled learners were students, and the percentage holding a post-graduate degree, Master's (33.0%) or Doctorate (29.9%), was above Coursera averages, 25.7 and 4.09%, respectively. The learners originated relatively more from Europe (32.3%), Africa (9.6%) and Oceania (3.1%), and less from Asia (24.9%), North America (22.7%) and South America (7.3%) compared to Coursera corresponding averages.

### Learning Objectives

The learning objectives cover the basics of metagenomics and the background knowledge necessary to consider the implementation of metagenomics in surveillance. They are enumerated for each MOOC module below, as published in the course platform^17^.

Module 1:

- “Distinguish between the concepts of metagenomics and other microbial genomics
- Give examples of the application of metagenomics
- Critique the need to use controls in different steps of a metagenomics study
- List types of controls that can be used in a metagenomics study
- Conclude on the advantages of metagenomics for the surveillance of antimicrobial resistance
- Evaluate how sampling design, sample size, sample material and sample handling influence the outcome of a metagenomics study
- Describe current sample processing for bacterial and viral metagenomics
- Explain different sequencing platforms and their possibilities regarding metagenomics
- Summarize the impact that library preparation may have on metagenomics results.”

Module 2:

- “Demonstrate the steps involved in a general bioinformatics analysis, including quality control and mapping to different databases
- Outline the principle behind various tools available for analysis of metagenomics data and explain the situations where each tool is appropriate to use
- Interpret the outputs of bioinformatics pipelines (read classification for antimicrobial resistance genes and bacterial species)
- Interpret the possibilities to use a database of antimicrobial resistance genes.”

Module 3:

- “Justify the need for epidemiology in surveillance
- Discriminate challenges for the use of metagenomics in surveillance
- Examine the potential of metagenomics for surveillance of pathogens and antimicrobial resistance
- Explain the concept of global and integrated surveillance
- Conclude on metagenomics findings together with explanatory data
- Employ methods for analysis and visualization of read counts.”

### Assessment

E-learning lecture- and quiz-specific feedback was retrieved from the trial run with volunteers. “The main outcome in terms of course improvement was the development of complementary reading material summarizing the content of the lectures, and the compilation of a glossary”^1^. Both were added to the revised e-learning version, before release as a MOOC. The Coursera platform offers several possibilities for learners' feedback. Module-specific feedback obtained from MOOC learners is presented in Table 1 including “likes” and “dislikes” given for each course element^1^.

“Additionally, an interactive voting tool^18^ was used during the workshop, at the end of each day, in order to collect feedback on both components of the training”^1^. 58% of all workshop participants had completed the e-learning and 7% planned to complete it after the workshop. 77% considered the blended learning more useful than a stand-alone e-learning or workshop. An online questionnaire was also used for the evaluation of the workshop and for collecting the participants' opinions on the workshop topic. Response rate was 80.5% (33/41 participants). Respondents assessed again positively the combination of the e-learning and the workshop, considering the workshop as an essential component of the training package. However, many would have liked to have longer practical sessions^1^.

At the time of writing (January 2020), the MOOC has run in 22 consecutive 4-weeks sessions, with a total of 3,346 learners enrolled, including 2,180 active learners (enrolled learners who have started a course item), of which 186 passed all assessments and were issued a course certificate. It has been rated as 4.7/5, with 95% of likes and 5% of dislikes. The highest drop rate among all eligible learners (81.9%) is in module 1. This is not surprising, as we expected most learners to explore the course content before deciding to complete it. Furthermore, it is in accordance with the 90-9-1 rule that describes most participation in online communities (90% consume content, 9% engage with content sporadically, and 1% regularly) (27).

## Discussion

### Lessons Learned

**Figure 1:**
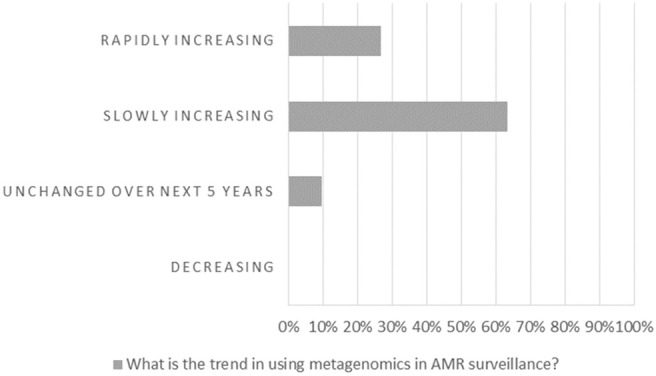
Workshop participants' opinion on the trend in using metagenomics in AMR surveillance.

**Figure 2:**
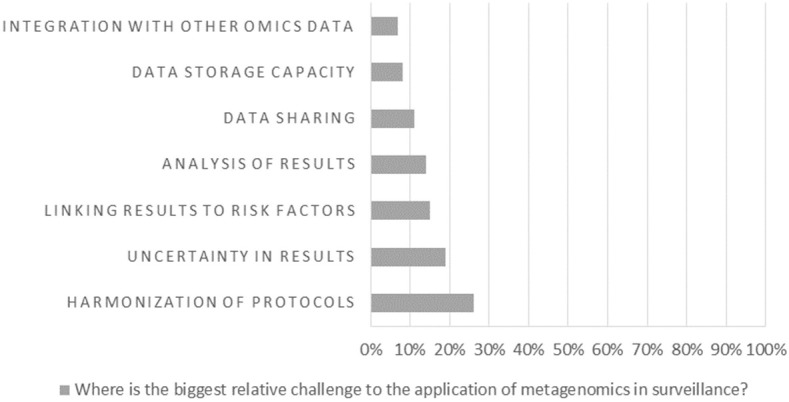
Workshop participants' opinion on the biggest relative challenge to the application of metagenomics in surveillance.

**Figure 3:**
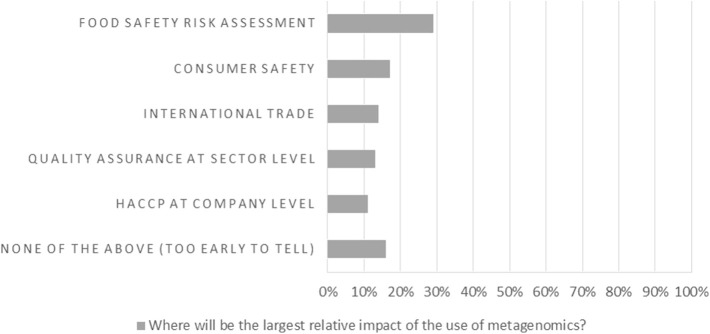
Workshop participants' opinion on where to expect the largest relative impact of the use of metagenomics.

At the end of the workshop, the majority of the participants (90.2%) responded that they expected the use of metagenomics in AMR surveillance to increase, slowly (63.4%) or rapidly (26.8%), in the near future (Figure 1). The participants were asked to assess the main challenges and gaps for the implementation of metagenomics in surveillance (Figure 2), and the results showed that harmonization of protocols and interpretation of results (including uncertainty and association of metagenomics data with risk factors) are considered main hurdles. The lack of standards and legislation, and the implementation costs were also mentioned. Infrastructure challenges, such as data sharing and storage were considered less relevant. Improvement of metagenomics analysis was also considered by the participants the priority in order to increase the understanding of AMR. However, the improvement of surveillance programmes and international guidelines, and an increase in harmonized reporting were considered similarly important (results not shown). Food safety risk assessment was clearly the area where participants considered metagenomics will have the largest impact (Figure 3).

### Practical Implications

The future of antimicrobial resistance surveillance needs to be tackled with a multinational, multidisciplinary One Health approach (1, 21). While many countries are already engaging in the use of whole genome sequencing for surveillance (9), outbreak investigation, source-attribution and microbial risk assessment (11), the implementation of metagenomics in those areas still resides in the future due to its novelty, among other reasons.

One of the main concerns about the routine use of metagenomics is that it may lead to a decrease in pathogen isolation from humans and along transmission pathways (9, 11, 28). However, the potential of metagenomics is significant. It allows the detection of pathogens in mixed cultures, the identification of (new) non-culturable pathogens, the characterization of bacterial diversity and its effect on pathogen presence and diversity, and the characterization of resistomes and mobilomes (sequences attributed to mobile genetic elements, involved in horizontal gene transfer). To engage in these diverse aspects of AMR surveillance and future methodological options, professionals from a variety of disciplines should co-develop a joint understanding of the strengths and weaknesses of this approach. Blended learning courses and MOOCs can be successfully applied in this context to deliver knowledge, to provide a platform to engage across disciplines, and to facilitate peer-learning.

The interaction with the course participants provided general information on the readiness of the community for using metagenomics in AMR surveillance. Harmonization of protocols was highlighted as an important challenge by the workshop participants. There is a general concern about the numerous sources of bias in metagenomics studies, and the need for validation and benchmarking exercises is recognized (11). Recently, there is a growing number of studies addressing this concern (29), which represent valuable input for a conscious application of metagenomics in surveillance. The lack of standards and legislation, lack of harmonized reporting and lack of international guidelines were also among the participants' apprehensions. Undeniably, metagenomics conveys sequence data that may contain indication of hazards which would otherwise not be investigated and/or detected with isolate-based monitoring methods. Additionally, host sequence data can potentially allow the identification of human subjects. These issues must be addressed in international guidelines developed for the ethical use of metagenomics (28). Improvement of metagenomics analysis was considered by the training participants the first priority in order to increase the understanding of AMR-related outputs (results not shown). “Improvement” may in this context relate to different factors that are considered potential limitations of metagenomics studies. One of the main challenges is that the detected DNA can originate from both dead and alive cells, which may be perceived as a shortcoming in the context of policy-based monitoring and risk assessment studies (11). Potential solutions could be to complement metagenomics with metatranscriptomics (28) or to use algorithms that infer microbial population replication rates from metagenomics data (30). However, the detection of non-viable microorganisms, particularly pathogens, may also be seen as an opportunity. Although dead bacteria may not constitute an immediate risk for the exposed population, their detection is an opportunity to prompt investigation of the source of contamination and to apply corrective preventive measures before transmission occurs. In a surveillance program, detecting the presence of pathogens (eventually carrying high-risk ARGs), viable or not, should therefore be desired–if the microorganisms are viable, their spread can be contained; if they are non-viable, source tracking can be performed and preventive measures applied to avoid infections. Also, attributing detected ARGs to their bacterial host, and classifying their transferability between hosts may be necessary in many circumstances. Metagenomic assembly and binning (31) help overcoming the first issue, and many recent developments have contributed to increase the number of genomes assembled from metagenomics datasets, including the methods of Hi-C Chromatin conformation capture (31), DNA methylation profiling (32) and co-assembly and co-binning (33). A greater challenge remains with the second issue-disclosing the link between ARGs and mobile genetic elements. The joint analysis of resistome and microbiome has been used to investigate the occurrence of horizontal gene transfer, with recent studies suggesting an infrequent exchange of ARGs between human gut flora and pathogenic organisms (34, 35). Another route to address this issue is the use of single cell sequencing (36, 37). A further concern is that the resolution in the profiling of resistomes, i.e., the accuracy of ARG typing, may be insufficient due to a high similarity shared between ARG reference sequences. This may produce ambiguous alignment, false negatives due to non-alignment, or false positives due to misannotation. Recent bioinformatics developments have also addressed this concern (35, 38). Similarly, the low sensitivity of metagenomics to capture low abundant ARGs, has also been recently addressed by combining targeted metagenomics with novel bioinformatics tools for the analysis of resistomes (39), however further developments and validation studies are still needed in order to confidently approach the sensitivity levels presently achieved with phenotypic methods.

Food safety risk assessment and consumer safety might benefit from metagenomics, in the participants' opinion. However, ARGs detected in metagenomics studies should undergo an assessment regarding their public health risk potential, since they do not all represent an actual hazard (2). The application of metagenomics in risk assessment is therefore dependent on a new hazard definition concept, and the nature of the hazard will determine the nature of the estimated risk. With metagenomics, “hazard” covers the microbial community, the resistome, and the potential for horizontal transmission of ARGs. As a result, risk may refer to the development of disease due to infection with a resistant pathogen, and/or the spread of ARGs between pathogens and commensal bacteria in the human host (40). Traditional microbial risk assessment methods need to undergo an adaptation in order to accommodate these new considerations of hazard and risk (40).

We developed and delivered a blended-training on “Metagenomics applied to surveillance of pathogens and AMR.” After the training, the e-learning component was revised and an updated version is now publicly available as a MOOC at https://www.coursera.org/learn/metagenomics^1^, on which more than 3,000 learners have already enrolled. The MOOC conveys the idea of the workflow, the requirements, the benefits and the challenges of AMR surveillance by metagenomics, which could help inform the design of future AMR surveillance programs.

### Constraints and Future Perspectives

Throughout the training, the main challenge has been to adjust to the variable level of background and skills of the participants. In general, the hands-on training was well-received, both during the workshop, and by the MOOC learners. However, when technical difficulties arise in operating the software programs for data analysis, it is difficult to provide adequate support to those in need. Furthermore, in the context of education at the global level, the uneven access of learners to infrastructures (internet bandwidth, computer processor, operating system and memory) will impact on the learning outcome and the likelihood of course completion. This mirrors one of the expected challenges in the implementation of a metagenomics-based global surveillance–the uneven and variable levels of capacity among countries.

A future perspective for improvement of the MOOC is to provide less technically demanding and infrastructure-dependent practical exercises. Furthermore, we intend to periodically review the course content and update it following the latest research developments. For example, many studies have recently investigated the impact of different normalization approaches for metagenomics data (41–43), a topic that has not been addressed in the current MOOC version. With future content updates, the course will maintain a high educative value and can be established as a reference international source of information for the implementation of metagenomics in surveillance.

## Data Availability Statement

All datasets generated for this study are included in the article/supplementary material.

## Author Contributions

TH and AD conceived the e-learning curriculum with input from PM, OL, SP, VB, JN, PK, LV, CP, RK, MH, and RH. AD coordinated the development of the MOOC. AD, TH, PM, SP, VB, OL, JN, PK, LV, CP, RK, MH, and RH developed and recorded the lectures for the MOOC. KS conceived the workshop curriculum with input from AD, PM, AB, RL, SS, LV, and PL. KS and VG coordinated the development of the workshop in collaboration with AD, and were responsible for advertisement, registration of participants and development of participant surveys. OL, PM, AB, RL, SS, LV, PL, and AD developed the lectures and practical exercises for the workshop. AD was in charge of overall direction and planning of the training and wrote the manuscript with critical feedback from all authors.

### Conflict of Interest

KS and VG were employed by the company SAFOSO AG. RH was employed by the company Intomics A/S. The remaining authors declare that the research was conducted in the absence of any commercial or financial relationships that could be construed as a potential conflict of interest.

## Footnotes

^1^Metagenomics Training Report. Available Online at: http://www.effort-against-amr.eu/page/metagenomics-training-report.php.

^2^EPI-Net: Epidemiologic network. Available online at: https://www.combacte.com/about/epi-net.

^3^Joint Programming Initiative on Antimicrobial Resistance (JPIAMR). Available online at: https://www.jpiamr.eu.

^4^Analysis of antimicrobial consumption and resistance ('JIACRA' reports). Available online at: https://www.ema.europa.eu/en/veterinary-regulatory/overview/antimicrobial-resistance/analysis-antimicrobial-consumption-resistance-jiacra-reports.

^5^Action at EU level: The new EU One Health Action Plan against Antimicrobial Resistance. Available online at: https://ec.europa.eu/health/amr/action_eu_en.

^6^Antimicrobial resistance–theory and methods. Available online at: https://www.coursera.org/learn/antimicrobial-resistance.

^7^Antimicrobial & Antibiotic Resistance Courses. Available online at: https://www.futurelearn.com/courses/categories/health-and-psychology-courses/antimicrobial-and-antibiotic-resistance.

^8^Antimicrobial Resistance in the Food Chain. Available online at: https://www.futurelearn.com/courses/antimicrobial-resistance-food-chain.

^9^Bacterial Genomes: Disease Outbreaks and Antimicrobial Resistance. Available online at: https://www.futurelearn.com/courses/introduction-to-bacterial-genomics.

^10^Genomic Data Science Specialization. Available online at: https://www.coursera.org/specializations/genomic-data-science.

^11^Whole genome sequencing of bacterial genomes–tools and applications. Available online at: https://www.coursera.org/learn/wgs-bacteria.

^12^Bacterial Genomes: Accessing and Analysing Microbial Genome Data. Available online at: https://www.futurelearn.com/courses/bacterial-genomes-access-and-analysis.

^13^Whole Genome Sequencing: Decoding the Language of Life and Health. Available online at: https://www.futurelearn.com/courses/whole-genome-sequencing.

^14^Bacterial Genomes: From DNA to Protein Function Using Bioinformatics. Available online at: https://www.futurelearn.com/courses/bacterial-genomes-bioinformatics.

^15^One Health: Connecting Humans, Animals and the Environment. Available online at: https://www.futurelearn.com/courses/one-health.

^16^Ecology from Farm to Fork Of Microbial drug Resistance and Transmission (EFFORT). Available online at: http://www.effort-against-amr.eu.

^17^Coursera. Available online at: https://about.coursera.org.

^18^Mentimeter. Available Online at: https://www.mentimeter.com.
